# Review of Learning-Based Robotic Manipulation in Cluttered Environments

**DOI:** 10.3390/s22207938

**Published:** 2022-10-18

**Authors:** Marwan Qaid Mohammed, Lee Chung Kwek, Shing Chyi Chua, Arafat Al-Dhaqm, Saeid Nahavandi, Taiseer Abdalla Elfadil Eisa, Muhammad Fahmi Miskon, Mohammed Nasser Al-Mhiqani, Abdulalem Ali, Mohammed Abaker, Esmail Ali Alandoli

**Affiliations:** 1 Faculty of Engineering and Technology, Multimedia University (MMU), Ayer Keroh, Melaka 75450, Malaysia; 2 School of Computing, Faculty of Engineering, Universiti Teknologi Malaysia, Skudai, Johor Bahru 81310, Malaysia; 3 Institute for Intelligent Systems, Research and Innovation, (IISRI), Deakin University, Geelong, VIC 3216, Australia; 4 Department of Information Systems-Girls Section, King Khalid University, Mahayil 62529, Saudi Arabia; 5 Faculty of Electrical Engineering, Universiti Teknikal Malaysia Melaka (UTeM), Melaka 76100, Malaysia; 6 Faculty of Information Communication Technology, Universiti Teknikal Malaysia Melaka (UTeM), Melaka 76100, Malaysia; 7 Department Computer Science of Community College, King Khalid University, Muhayel Aseer 61913, Saudi Arabia

**Keywords:** robotics, robotic manipulation, object manipulation, object grasping, deep reinforcement learning, dense clutter, cluttered environment, sensory data

## Abstract

Robotic manipulation refers to how robots intelligently interact with the objects in their surroundings, such as grasping and carrying an object from one place to another. Dexterous manipulating skills enable robots to assist humans in accomplishing various tasks that might be too dangerous or difficult to do. This requires robots to intelligently plan and control the actions of their hands and arms. Object manipulation is a vital skill in several robotic tasks. However, it poses a challenge to robotics. The motivation behind this review paper is to review and analyze the most relevant studies on learning-based object manipulation in clutter. Unlike other reviews, this review paper provides valuable insights into the manipulation of objects using deep reinforcement learning (deep RL) in dense clutter. Various studies are examined by surveying existing literature and investigating various aspects, namely, the intended applications, the techniques applied, the challenges faced by researchers, and the recommendations adopted to overcome these obstacles. In this review, we divide deep RL-based robotic manipulation tasks in cluttered environments into three categories, namely, object removal, assembly and rearrangement, and object retrieval and singulation tasks. We then discuss the challenges and potential prospects of object manipulation in clutter. The findings of this review are intended to assist in establishing important guidelines and directions for academics and researchers in the future.

## 1. Introduction

Humans’ ability to manipulate objects with little or no prior knowledge continues to be a source of inspiration for robotics researchers. The skill of manipulating objects is involved in a variety of robotic applications, from package sorting at a logistics center to bin picking in a factory. In order for a robot to interact with its environment, it needs to be able to perceive its surroundings. This gives the robot the information it needs to decide what an object is and where it is. Traditional robotic manipulation strategies depend on prior object knowledge, such as computer-aided design models and 3D objects, to estimate grasp pose [1]. These systems are vulnerable to mistakes when dealing with novel objects in cluttered situations since they may be inaccessible. 

In the near future, robots are anticipated to be integral parts of our everyday lives as our friends at home and at work. In order to meet this expectation, robots will need to be able to do everyday manipulation tasks, which are important for most household tasks such as cooking, cleaning, and shopping. Robots have been used successfully in factories for many years to do tasks that require manipulation. Designing skills for robots that work in dense cluttered environments can be hard in many different areas, such as computer vision, automated planning, and how humans and robots interact.

As described in [2], the sensing approach, the learning approach, and the gripper design approach are all used to tackle manipulation challenges in robotics. Each has made a significant contribution to robotic performance by executing basic to sophisticated tasks. Several studies [3,4,5,6,7] have highlighted sensory approaches to improve gripping. Some studies have looked at how tactile and vision sensors may develop robotic technology by extracting internal (tactile sensor) and external (vision sensor) object features. According to these studies, a robot must be able to perceive and interpret its environment through sensory capabilities. Other investigations found the sensory technique unsatisfactory if the grippers are poorly constructed. Designing grippers helps improve robotic grasping’s sensory approach. Design of grippers, including rigid parallel-jaw finger and multi-finger grippers [8] and soft grippers [9,10,11], has been addressed extensively. Many researchers have employed different materials to construct rigid and soft grippers to support the idea that sensory information and gripper design work together [12,13]. Sensory and gripper design approaches have been studied for a range of difficulties.

The learning approach includes computer vision or cognitive learning, which is necessary for robots to operate intelligently in human environments and tackle whatever circumstances that may arise. Thus, robots are taught to work with people and help them with a wide range of everyday tasks [14]. When these two machine learning techniques are combined, a new field called “deep RL” is created, which is a subset of machine learning. In deep RL, the power of deep learning can be used to solve the reinforcement learning (RL) problem due to the limitations of Q-tables, which can be less efficient in robotics because of the huge number of states. Thus, the deep RL framework employs deep neural networks to map the states (perceptual input) into action values (*Q*-value or Q-function). RL then takes that action value and performs the corresponding action. This action is evaluated through a loss function via backpropagation to update the weights in the networks using the particular optimizer. When this is used for robotic manipulation, the robot looks at the environment through sensors (such as cameras and touch sensors) and tries to take the best action based on a policy that has already been predefined. One of the learning strategies that has recently been used in robotics is the deep RL framework, in which an agent interacts with the environment to learn the optimal policy via trial and error.

Robotic manipulation can be performed in different circumstances for different purposes that have been addressed in different studies [15]. For example, deep RL approaches have been used to assist robots in performing sophisticated robotic manipulation tasks in various applications, such as deformable object manipulation [16], heavy object manipulation [17], and pick-to-place tasks [18,19,20]. Even though several studies have focused on the learning approach (e.g., deep RL) to solve robotic manipulation problems, it still requires further studies, as stated in [21]. This review paper focuses on reviewing and analyzing deep RL-based robotics manipulation challenges in a cluttered environment. This is in contrast to other reviews, which discuss the current state of deep RL-based robotic manipulation in different areas, such as robotic manipulation [22], robotic grasping [21], pick-and-place operations [23], production systems [24], and bin picking approaches [25]. Object manipulation in cluttered environments continues to be a major unaddressed challenge, despite the enthusiasm of the scientific community and its practical importance. In this review paper, the learning approach-based robotic manipulation in dense clutter is chosen as the domain of study review that requires more investigation.

This review paper aims to draw attention to the variety of object manipulation issues that these approaches were applied to and propose potential directions for further study. This paper discusses problems with robotic manipulation in environments with dense clutter from a cognitive point of view. It also discusses recent published works and cutting-edge approaches to solving these problems, as well as open problems that must be solved in order to overcome manipulation challenges in cluttered environments. The main contribution of this review paper is to explore appropriate cutting-edge learning algorithms and how they can be used in addressing robotic manipulation challenges in a cluttered environment. This will help researchers deal with important research questions about robot performance and make important recommendations for researchers and practitioners in the future. We believe that researchers in the field of robotics will find this review to be a useful tool.

This review paper starts by giving a brief background on the domain of learning-based robotics and pointing out the important review articles in this domain. An explanation of the terminology aspects of RL is described in Section 2. Then, in Section 3, the review protocol methodology used in this review paper is explained. Section 4 presents the numerical analysis of the final set of articles, including the number of final sets of articles per database and the numerical analysis of the final sets of articles with the related tasks. We examine and discuss in depth three categories of related studies that concentrate on object manipulation in clutter in Section 5. The challenges of the current studies are then addressed in Section 6 as a direction for the future. Section 7 then proposes a recommendation that might be considered to enhance the existing methods in robotics. The paper then concludes by outlining the main concepts that are covered in this review paper.

## 2. Essential Reinforcement Learning Terminologies

Current research has shown how deep reinforcement learning helps a robot perform a certain robotic manipulation. Thus, there is some terminology that needs to be defined in the reinforcement learning framework. Multiple references have discussed these terms [26,27], which are fundamental to comprehending reinforcement learning for any researcher in this field and are considered important to be understood before reading any research publication. However, in this part, we attempted to simplify these terms so that they can be clearly understood, as listed in Table 1.

## 3. The Review Protocol Methodology

The review search was conducted on three distinct digital datasets. The articles were chosen on the basis of an index that supports both simple and complex search queries. They have been published in a number of journals and conference papers on the topic of learning-based object manipulation amid dense clutter. In this review, we considered the engineering and computer science disciplines as the most important criteria in learning-based robotic manipulation. Review searches were carried out on the following three digital databases: (1) IEEE Xplore (IEEE), (2) ScienceDirect (SciDir), and (3) Web of Science (WoS). Thus, the research selection process entailed a comprehensive search for relevant articles that was divided into two parts:

- Duplicated and irrelevant papers were eliminated by scanning the article titles and abstracts.
- The full contents of the articles that were filtered out in the first part were then read, and the articles were classified into taxonomic groups.

In terms of search queries, the advanced search settings on all the search engines excluded book chapters and other documents, but they included journal articles and conference papers written in English. In addition, full-text articles and conferences published between 2016 and 2022 were considered for review. For the purpose of searching all of the databases that were specified, the following keywords were utilized: (“Manipulation” OR “Grasp” OR “Grasping” OR “Pick” OR “Robotic”) AND (“Reinforcement Learning” OR “Self-Supervised Learning”).

Current research themes revealed by the most recent literature are highlighted and discussed in this study, with a focus on the most significant challenges. Using well-known digital database sources where the majority of robotics journals are indexed, we intended to review hundreds of papers over the last seven years. For the benefit of future researchers, we wanted to provide a comprehensive overview of prior research as well as an impression of existing challenges. These three databases were selected because they include the vast majority of relevant literature on learning-based robotic manipulation. All significant journals in this field are indexed in one of the three aforementioned databases. In addition, practically every journal mentioned in these three databases is also indexed in Scopus. We covered articles from the last seven years, between 2016 and 2022. This period is regarded as the most productive time for intensive study of this review subject, and a rise in research occurred at this time. 

## 4. Numerical Analysis of Final Set of Articles

**Figure 1:**
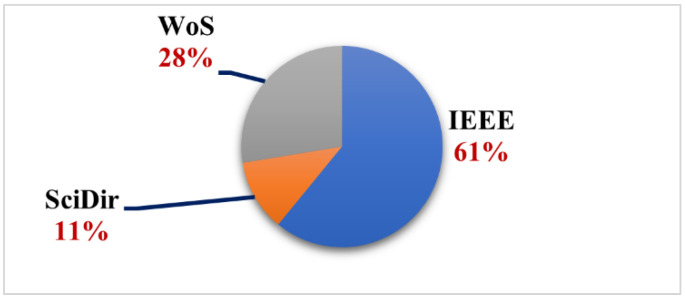
Number of final sets of articles per database engine.

We believe that by giving a numerical analysis of the final collection of papers, researchers can have a better understanding of the importance of the grasping in clutter challenges discussed in this critical review study. As mentioned in the critical review process section, three databases were chosen on which our research query was performed. The number of articles retrieved from each source varies, as seen in the pie chart in Figure 1. The IEEE database accounts for almost more than half of the final set of articles, followed by WoS, which takes up roughly a quarter. Such an aspect of the study could help researchers in selecting where to submit their works because the most relevant articles in this field are indexed in IEEE and WoS.

**Figure 2:**
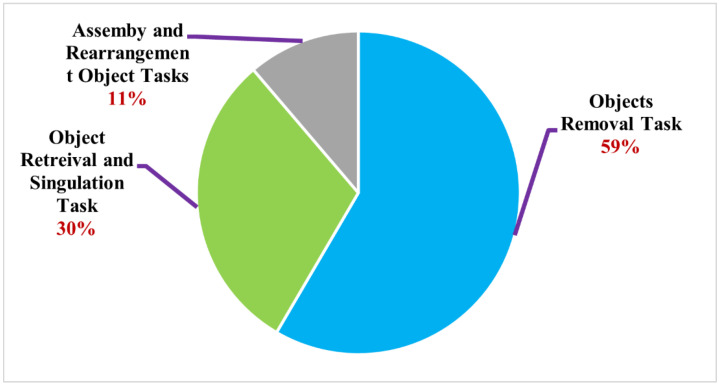
Numerical analysis of the final set of articles with their associated tasks.

Figure 2 shows that object removal tasks account for more than half of all articles, followed by object retrieval, singulation tasks, and assembly and rearrangement tasks. Accordingly, the majority of studies concentrated on executing grasp to clear objects off a tabletop. It indicates that many approaches have been used in training robots to do so. This type of task seems simple, yet it remains a challenge. In terms of learning policy, the other taxonomy tasks appear to be more sophisticated when compared to the objects removal task, which may be the critical stage of robot learning. It would be interesting to answer the question, “Why are half of the articles concentrating on objects removal tasks?” If we dive further into the studies, it can be seen that the clearing object task does not merely involve a single-grasp action strategy. Instead, various techniques have been attempted to address challenges in various cluttered scenarios by developing reliable and efficient learning approaches. In the next section, we thoroughly examine and evaluate the related studies in each taxonomic category of the final set of articles.

## 5. Critical Review 

Recently, many researchers have incorporated deep reinforcement learning (RL) in effective robotic grasping, particularly in cluttered environments. Significant progress has been reported in this field; however, deeper understanding of the problem and more investigation on new learning policy are needed to further improve the grasp success probabilities. The succeeding discussions critically examine the related papers in this domain and emphasize the existing issues of object manipulation in a cluttered environment.

**Figure 3:**
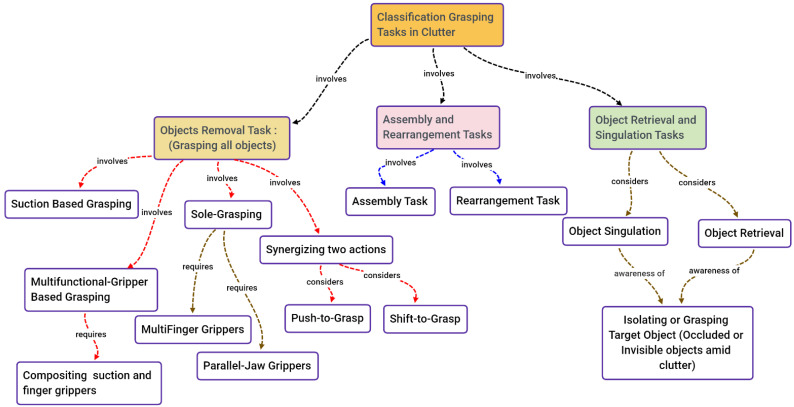
Taxonomy of the final set of articles.

Many studies have been investigated through a review of the literature and an examination of various elements, such as the intended applications, the methodologies used, the difficulties experienced by researchers, and the recommendations adopted to overcome the challenges. Grasping in cluttered environments has been widely investigated because of the papers’ various contributions in the last seven years. In this review, the papers in the literature about object manipulation in cluttered environments are put into three groups. As illustrated in Figure 3, there are three types of tasks: (1) object removal tasks; (2) object retrieval and singulation tasks; and (3) assembly and rearrangement tasks.

### 5.1. The Removal of Objects TASK

**Figure 4:**
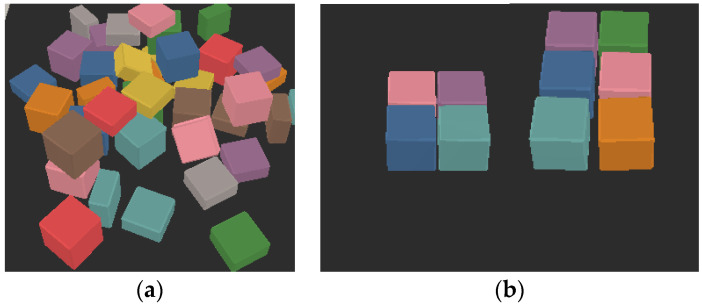
Common object manipulation scenarios in clutter: (**a**) random cluttered scenario (RCS); and (**b**) well-arranged scenario (WAS).

The process of teaching a robot to grasp and remove objects from its workplace in a crowded environment is known as object removal. In order to conduct a series of actions on its surroundings and objects, the robot must be able to perceive and detect them. In scenarios where objects are physically close together, the robot’s gripper must find a place for its fingers to perform the grasping action. For instance, engaging with scenarios including densely cluttered objects seems to be a challenging grasping task in robotic manipulation. In such scenarios, the robot arms must use a skillful learning strategy to successfully complete a grasping operation. Researchers have focused on two situations: objects that are placed in random clutter (Figure 4a) and objects that are placed in well-arranged clutter (Figure 4b). Both scenarios have been carefully examined, and they are even regarded as the most difficult tasks, needing a variety of learning strategies. 

**Figure 5:**
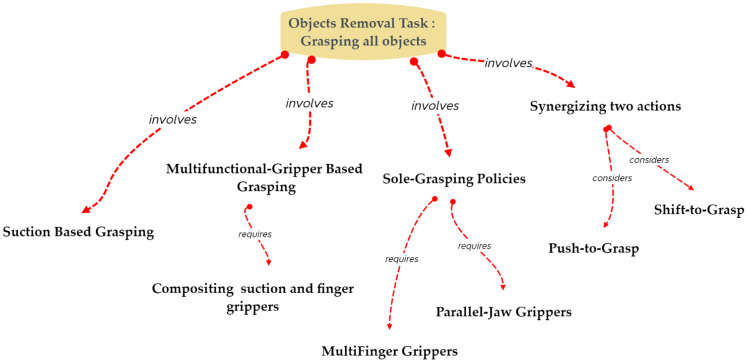
Strategies implemented in object removal task.

In order to make it easier to remove objects from cluttered environments, sole-grasping policies, suction-based grasping, multifunctional gripper-based grasping, and two-action synergy have all been employed, as illustrated in Figure 5. These four strategies and their associated works are thoroughly discussed.

The problem of sole-grasping policy, for example, has been extensively addressed, either by offering dataset-based object detection or by enhancing the learning framework. However, in certain situations, using sole grasping may not be effective. Instead, suction grasping has shown promise as a way to improve the performance of robotic manipulation, and a number of publications have been published to solve problems that grippers might have. Another strategy is to combine a parallel-jaw and suction mechanism into a single gripper that can assist in addressing issues that the sole-grasp and suction-grasp policies are unable to address. The three mechanisms mentioned above can perform robotic manipulation tasks efficiently in certain scenarios, but they are less accurate in others. Learning to grasp by coordinating two actions, such as “push-to-grasp” or “shift-to-grasp,” can be helpful in situations where the objects are well-arranged. Each mechanism aims to resolve a specific issue related to the challenge of a cluttered environment as it learns to manipulate objects in clutter via the use of supervised learning approaches. Despite extensive study on the topic, the task of removing objects is still challenging.

#### 5.1.1. Sole-Grasping Policy

The sole-grasping policy is an approach for training a robot to only use the grasp action to grasp objects when no other actions (e.g., pushing, shifting, and poking) are involved. Although many studies have concentrated on learning to grasp a single object or multiple objects, some of these studies have focused on overcoming the difficulty of grasping in clutter, where objects seem to be stuck on one another in a pile. In this case, the robot must be able to effectively perceive and interpret the objects and environment to clean the objects from its workspace. For example, Pajarinen and Kyrki [28] proposed a partially observable Markov decision process (POMDP) to avoid heuristic greedy manipulation; however, it is inadequate for unknown multi-object manipulation in cluttered scenarios. Their approach achieves global reward-based optimization despite temporal uncertainty and incomplete data. Their POMDP approach may automatically adjust object-specific action success probabilities and occlusion-dependent observations and actions. Emerging POMDP solutions can handle huge state and observation spaces, but not action spaces. Because geometric constraints and robot mechanics are not addressed, this strategy is overly optimistic.

In [29], an Accept Synthetic Objects as Real (ASOR)-based data augmentation strategy was presented to enable the generation of training data from demonstrations gathered in clutter-free situations, which is effective for manipulation in cluttered surroundings. By encoding the characteristics of attention, two network topologies—implicit attention (ASOR-IA) and explicit attention (ASOR-EA)—are used to create spatial attention on a target object and its corresponding motor component. Two network models and data augmentation are utilized to train robot controllers in an end-to-end manner that can be performed in the densely cluttered environment. However, this study is restricted to the training data since it learns from demonstration.

The interest in robots with warehouse automation abilities has significantly increased in recent years due to the fact that robots are often successful at handling a variety of objects. To address that, several studies have trained deep RL algorithms with RGB-D data, which is being utilized to enhance robotic vision-based grasping in cluttered environments. For example, RGB-D data from multi-view-based data-driven learning has been proposed by Zeng et al. [30] to address bin picking. They trained Q-leaning on the fully convolutional network (FCN). In their approach, they segmented and labeled several views of the scene, and then fitted the pre-scanned 3D object models to the segmentation to create a 6D target object. The aim of their approach is to minimize the manual effort in generating data and enhance the accuracy of object detection using bounding-box theory. In [31], “Grasping in the Wild,” which allows 6D grasping of unknown objects based on gathering grasping demonstrations by individuals in a variety of situations, was addressed. Q-learning was utilized to learn the optimal Q-function on the basis of minimizing the temporal difference errors between the present *Q*-value and the goal. 

In addition, robotic grasping in clutter has been addressed [32] on the basis of the deep RL context by training Q-learning on DenseNet, which is a fully connected layer and a pretrained model on ImageNet. Instead, the same author proposed a color matching-based approach [33] that consisted of two parts. The first part was a semantic segmentation module, which was used to segment the color image and generate a mask of the intended target object in order to identify the target object. The action was performed in the second part, using the Q-learning framework that evaluated the action’s performance. 

In [34], dense object nets, valuable visual object representations that can be learned only by robot self-supervision, were introduced. The purpose of their study was to develop an approach for robotic manipulation in clutter that is built on learning pixel-level data correlations, for object-centricity, multi-object distinct descriptors, and learning dense descriptors. Due to the large number of object classes, each could not be divided into independent areas of descriptor space; hence, only a thick descriptor was supplied. Their approach also needs a human user to specify graspable points in the scene. Similarly, Song et al. [35] used depth image data to create a real-time deep convolutional encoder–decoder neural network (NN) for robotic grasping in clutter. The U-grasping fully convolutional neural network (UG-Net) can estimate grip quality and posture pixel-by-pixel using depth images. The network inputs the RGB-D camera’s depth image and outputs each pixel’s grab quality, location, and gripper width. Inaccurate z-axis coordinate predictions and overestimated gripper breadth caused failed grasps. The grasping issue in clutter was addressed by Chen et al. [36]; this issue is challenging for robot vision systems owing to partial object occlusion because a vision system could be less efficient in object detection. Chen et al. employed RL and RGB-D cameras to obtain graspable points. The modular pipeline was compensated by an object detector, perspective optimizer, and grip planner using binary segmentation masks and high-level instructions. For image training, they created a large-scale multi-view real-embodied dataset (RED) as a data-driven simulator. Even though a portable active grasping tool was used to optimize views for successful grasping in clutter, modal segmentation is better than the binary segmentation mask. 

Numerous studies have mostly used RGB images without depth data. Using the ability of humans to manipulate target objects, a single set of RGB data has been leveraged with generative adversarial networks (GANs) [37] to predict the hand robot’s position and shape while conducting grasping on multiple objects. Despite GANs’ versatility, training was unstable and needed hyperparameter adjustment. Furthermore, the grasp point in relation to the desired action and state of an object has not been considered. An RL-based grasp pose detection dataset was created by Kalashnikov et al. [38] by employing RGB data for training high-accuracy control policies for picking objects in cluttered environments.

In contrast, reference [39] addressed the challenge of sim-to-real fidelity gaps and the high sample complexity of on-policy RL algorithms. Initially, RL was adopted to train a robotic arm with a multi-finger gripper to obtain the optimal policy during grasping object tasks in clutter via simulation. Their method worked in the context of the pixel space of the input (e.g., a single-depth image). A depth-image-based mapping from pixel space to Cartesian space increased grasping likelihood in cluttered scenes. However, the dependence on external planners to develop alternative grasps and the grasping input representation in ANNs prevented the implementation of deep learning for multi-finger gripper-based grasping. The first issue is that when a robot is in operation, it may come into contact with a large variety of objects, each of which may need a different configuration of a multi-finger gripper. Therefore, learning to grasp an object necessitates representations that are capable of effectively handling both the geometry of the object and the grip configuration, which are necessary to assess the success of the grasp in terms of the data and computation associated. The second issue is caused by the increased search complexity that is necessary to arrange multi-finger grasps in comparison to that which is needed for the parallel-jaw grasp.

Many studies have attempted to solve the problem of grasping objects in dense clutter due to the growing demand to learn 6 degrees of freedom (DoF) grasping [40,41]. An end-to-end network that efficiently distributes 6-DoF parallel-jaw grasps from a scene’s depth data has also been proposed [42] by proposing the 6-DoF grasps to be projected into a 4-DoF grasp representation, which is composed of 3-DoF grasp rotation and grasp width. Moreover, the challenge of 6-DoF grasping has been addressed by the utilization of geometrically consistent space and undistorted depth images [43]. Even though it was able to execute grasping effectively in the chaotic environment, it was occasionally unable to grasp the objects that were aligned with the edge of the box. Thus, as the environment becomes more complex, it becomes more difficult to grasp multiple targets in a cluttered or even dense environment.

Planar manipulation based on 4-DoF has proven to be effective in enhancing bin-picking tasks. Planar grasping, on the other hand, limits the robotic arm’s movement (e.g., up-down), which restricts its reachable positions, especially when the robotic arm is involved in performing bin picking. This problem was handled by Berscheid et al. [44] by keeping the component of planar grasping that is learned and adding a model-based controller to calculate the other two degrees of freedom (2 DoFs) to learn 6-DoF grasping. They employed a model-based controller to change the grasping primitives’ orientation during real-world training. Replacing a learned model with a model-based lateral controller trained on analytical measures could improve their method. In [45], the challenge of grasping in a confined environment (such as walls, bins, and shelves) was addressed (as it was in [44]), but the grasp pose’s reachability required a lot of consideration to prevent it colliding into structures. To address this issue, a study [45] proposed the CARP technique, which is “a collision-aware reachability predictor.” CARP could learn to assess the possibility of a collision-free grasp position, which would significantly improve grasping in challenging situations with a parallel-jaw gripper. In a similar way, robots in a dense environment must be able to pick up any object. In a number of current methods to grasp objects from dense environments, parallel-jaw grippers are used. However, these grippers are unable to carry out a range of robotic grasping tasks. Corsaro et al. [46] proposed that enhancing the grasp success probability of various kinds of grasps can be achieved by training multi-finger grippers based on a data-driven technique using a point cloud that was generated from the depth sensors. 

The generative attention learning (GenerAL) approach was proposed for 6-DoF grasping by leveraging deep RL to directly output the final position and configuration of the fingers [47]. Although the challenge of high-DOF grasping has been addressed by extending the pixel-attentive multi-finger grasping algorithm [39] to a complete generic framework that can be applied to robotic hands with arbitrary degrees of freedom, there are certain failure situations in clutter situations due to the highly dense cluster where there is no space for the robot to put its fingers. The GenerAL approach has not been concentrated on establishing object-specific grasping, and it requires a simulation setup and, in most situations, an extensive parameter search to operate sufficiently. To overcome the challenge of increasing computational time that existed in [47], the generative deep dexterous grasping in clutter (DDGC) has been proposed to generate a set of collision-free multi-finger grasps in cluttered scenes [48]. However, high-quality grasps produced by DDGC do not always give a successful grasp in reality. To address the challenge of discontinuous sampling of grasp candidates and lengthy computation times, generative grasping (GG)-CNN [49] was proposed to extract pixel-by-pixel the grasp quality from a depth image. Meanwhile, the optimal grasp was predicted by considering the position, angle, and grasping width. However, (GG)-CNN failed to grasp objects due to inaccurate visual information, as the depth camera could not adequately identify the objects in a high-clutter environment. In addition, (GG)-CNN failed to execute grasping on a black or transparent object in the middle of a cluttered environment. 

In summary, object grasping in cluttered environments using the sole-grasping policy has been reported in several studies. Some of the studies addressed the problems of generating high-DoF grasp poses by using multi-finger grippers to estimate the diversity of grasping points. Other studies have attempted to overcome the issue of the grasping of objects in a cluttered environment by including parallel-jaw grippers, which do not require as much space to function in cluttered spaces as multi-finger grippers. A few more studies have focused on creating efficient datasets to improve the grasp of robots in cluttered environments. Datasets may be used to perform either 2-DoF or 6-DoF grasping in cluttered environments, and the scheme can be executed with parallel-jaw or multi-finger grippers. Table 2 summarizes the relevant works in this domain, including the applied methodology, the purposes, and each method’s drawbacks.

#### 5.1.2. Suction-Based Grasping

Suction grasping is another mechanism strategy has been involved in performing object manipulation in dense clutter. In certain circumstances, such as when a finger-gripper struggles to grasp an object in a cluttered environment, a mechanism that relies on suction-based grasping is thought to be an effective alternative. Industrial and warehouse demand fulfilment employs suction grasping for pick-and-place tasks. As seen in the “Amazon Picking Challenge [54]”, suction can reach into narrow areas and pick up objects with a single point of contact. However, suction-based grasping has attracted less study compared to parallel-jaw and multi-finger grasping. This is because suction grasping requires dexterous grasping, which necessitates intelligent visual observation to accurately predict the grasp point of the target objects to avoid grasp failure when they come into contact with an object’s edge or a non-vertical surface. Moreover, suction grasping is limited to certain types of objects, which means it cannot be used for other types. This section reviews the papers that are associated with suction-based grasping in clutter. Instead of learning to use a finger-gripper, the focus is on suction as a solution.

Mitash et al. [55] proposed implementing point cloud segmentation, which yields a more reliable grasp point than semantic segmentation. Their purpose was to improve the probability of grasp success rate for suction grasp utilizing the dataset of Zeng et al. [30]. On the other hand, their approach takes a long time to finish a multi-trial since it performs grasping tasks with a single arm. Thus, training dual-arm robots to work cooperatively can avoid the time-consuming part, thus eliminating the challenge of the single-arm limitation. Kitagawa et al. [56] devised a multi-stage learning technique for selective dual-arm grasping that uses CNN-based semantic segmentation to predict grasping points. Automatic annotation allowed the network to predict grasping points in the first stage. For both single-arm and dual-arm gripping movements, the robot could discover novel grabbing positions and eliminate ineffective ones as it acquired experience. They focused on grasp point prediction and implemented CNN-based automatic grasp point annotation learning. Their annotation process, on the other hand, was performed by hand, which made it less reliable than the other methods.

In [57], suction grasp was proposed as an alternative solution to manipulate objects in a cluttered environment to alleviate some of the failure situations that may be caused by the pushing behavior as a consequence of synergizing the push and grasp operations. Their framework entailed deep RL (e.g., training Q-learning with ResNet and the U-net structure). However, their framework was validated using CoppeliaSim (V-REP) simulation. Moreover, suction grasp points were predicted randomly, which makes it hard for their framework to predict exact grasp points in dense clutter. Han et al. proposed an approach that is an object-agnostic approach for detecting suction grasp affordance [58]. In their approach, the graspable object area is predicted using a suction grasp point affordance network (SGPA-Net), whereas graspable points are predicted using a fast-region estimation network. However, the two networks were trained with mixed loss functions, which yielded disappointing results. There is also a flaw in their approach, which is associated with the vacuum suction pad. For example, it was unpredictable when it came into contact with an object’s edge or a non-vertical surface. In another study [59], RL with dense object descriptors was proposed for performing grasping tasks in a cluttered area.

#### 5.1.3. Multifunctional Gripper-Based Grasping

Another mechanism that has attracted the attention of several recent research studies is the training of RL to coordinate the execution of grip and suction grasp. The implication is that the robotic arm is outfitted with a gripper that can perform both finger-gripping and suction-cup functions. This multifunctional gripper design enables exploiting the power of the finger-gripper in executing grasping in clutter to overcome the suction-cup restriction, and vice versa, in one gripper. For example, the study [60] has proposed learning the robotic manipulation of objects (e.g., pick-and-place objects) by predicting both grip and suction affordance using a multifunctional gripper. In their approach, the suction affordance was predicted using a fully convolutional residual network for each multi-view RGB-D image. A category-agnostic affordance prediction technique was used to choose and execute one of the four potential grasping primitive behaviors. However, planar grasps have also been executed within their learning approach, which might face difficulty due to arm movement restrictions.

The difficulty of grasping tiny and light objects has not yet been thoroughly studied in bin-picking scenarios. In this situation, pre-grasp actions are required since spatial grasping cannot be avoided in a densely structured clutter. An object can be spatially grasped in clutter by wrapping a continuum manipulator around it and squeezing it. Alternatively, the manipulator could perform push or shift actions to explore the environment for easy grasping, which is called a pre-grasping operation. For instance, the DQN has been proposed to coordinate the grip and suction gripper-based dexterous actions using the affordance map [61]. Their approach was aimed at assisting the robotic hand to actively explore the environment until the optimal affordance map is obtained. This study [61] was adapted from their previous works [62,63]. However, they implemented tactile sensing in [61] as a way to enhance the grasping performance and improve grasp efficiency; in addition, more experimental tests were provided. Although their proposed approach significantly enhanced the robotic manipulation in clutter, an inefficient push was performed [61], which could not make a change to the robot workspace because their approach needs more training time to acquire the optimal learning policy. In addition, the suction grasp failed in some situations where the suction graspable point was detected on the edge of the object, which led to object slippage.

Another study proposed the idea of “see to act,” which implies that a robot learns to perceive and then transfers that knowledge to learn to act [64]. The affordance model was utilized to generate the affordance in a pixel manner. Thus, each pixel has a predicted affordance value, which represents the complete effectiveness of the related actions at the 3D location. Based on the maximum affordance value, the robot then performs robotic grasping either using a finger gripper or suction cup. Furthermore, planar grasping was implemented, which could fail in some situations where the objects are aligned with the wall or in dense clutter since their approach was tested with less challenging clutter.

#### 5.1.4. Synergy of Two Primitive Actions 

Fundamental movements involved in prehensile (e.g., grasping) or non-prehensile (e.g., pushing, shifting, and poking) activities are known as primitive actions. The policy learning of making prehensile and non-prehensile movements work cooperatively is the way to perform the robotic task of detaching the object arrangement in clutter. For example, the push action can be synergized with the grasp action to work as a complementary part to achieve the robotic task in clutter. Several studies have dedicated this learning policy to addressing the challenges of learning robots to detach the objects arranged in clutter. 

The synergizing of two actions is formulated as a Markov decision process. The agent (e.g., robot) chooses and performs an action in accordance with a policy $\pi \left(s_{t}\right)$ in any given state $s_{t}$ at time t, then moves to a new state $s_{t+1}$ and gets an immediate corresponding reward $R_{a_{t}}\left(s_{t},{\;s}_{t+1}\right).$ Finding the optimal policy $\pi^{*}$ ($\pi^{*}:S\to A$) based on maximizing the expected total of future rewards, provided by $R_{t}=\overset{T}{\underset{i=t}{\sum }}\gamma^{i-t}\;R_{a}{}_{i}$, that is, the discounted sum across an infinite horizon of future returns from time t to ∞, is the objective of our robotic reinforcement learning issue. The most common existing strategy of synergizing the two actions (Ψ) is to predict the grasp point and push direction on separate neural networks (∅), and then select the max *Q*-value of either to grasp (g) or to push (p) be executed at the 3D location generated from a pixel (ρx) as express in the following equation [65]:$\underset{a_{t+1}}{argmax}\left(Q\left(s_{t},\;a_{t+1}\right)\right)=\underset{\left(\Psi ,\rho x\right)}{argmax}\left(\emptyset_{p}\left(s_{t}\right),\emptyset_{g}\left(s_{t}\right)\right)$.

The study in [66] addresses the challenge of grasping objects once they are close to each other, where there is no space for gripper fingers to perform grasping. The notion of their approach is to predict the grasp point and push direction using separate fully convolutional networks (FCNs). Then, the highest *Q*-value is selected, and the corresponding action is performed using Q-learning. Another study [66] proposed a rule-based method to synergize push and grasp actions that leverages deep RL. However, rule-based approaches are less effective because the robot may keep pushing once the push actions are performed without making any changes to the robot’s workspace, which affects the robot’s performance.

The majority of the environments mentioned in the literature review have a loose set of restrictions regarding object configuration and available pushing actions, as opposed to the settings in constrained environments (shelves or corners of bins), where only a small number of pushing actions are permitted. It is interesting how many studies have tried to tackle this problem. Another strategy for enhancing object grasping amid clutter is shifting objects [67], which involves putting a finger on top of the target object to increase grasp probabilities. The approach in [67] leverages applying thresholds to synergize the shift and grasp actions (e.g., $\psi_{g}$ is a threshold probability, choosing between the grasping and shifting effort, and $\rho_{s}$ is a threshold between the shift attempt and the assumption of an empty bin). Although $\psi_{g}$ may be regarded as a high-level parameter to indicate the system’s cautiousness against grasp attempts and its ability to increase the chance of a successful grasp, the resilience to missing depth information is a crucial component for improving the robotic bin grasping process, as the depth availability of stereo cameras is restricted by shadows or reflecting surfaces. When an object is near the totebox’s edge or even at its corner, the grasping of this object is challenging. Moreover, when objects are piled together, there may be no grasps for the robot to choose from. To deal with these situations, the study [68] combined the grasping and pushing actions by using a deep RL approach. They advocated combining the enhancements to the grasp’s quality $Q_{g}$ with an obtaining strategy to achieve a pushing action to ensure that the push will enhance the grasp availability. A twofold experience replay is also proposed to enhance the search for totebox borders. The standard strategy depicted by $a_{t}=argmax\left[Q_{p}\left(s_{t}\right),\;Q_{g}\left(s_{t}\right)\right]$ is enhanced by utilizing the notion of thresholds for grasp $\tau_{g}$ and push $\tau_{p}$ to achieve a compromise between efficiency and robustness. 

Yang et al. [69] proposed training the Q-Learning algorithm with an Attention Deep Network, and the paper referred to this as deep reinforcement learning due to the combination of a deep learning model (Attention Deep Network) and reinforcement learning (Q-learning). In their work, they used two parallel-trained network branches with the same structure for pushing and grabbing. It is almost identical in principle to [65]’s strategy learning approach (which was retrained and tested from scratch by us [70]). There is, however, no significant difference, except they added Attention Network after DenseNet to improve the network model’s performance by adding a channel attention mechanism to weight the DenseNet feature channels. Due to their reliance on the max-value to synergize push and grasp, excessive pushing of objects (out of view) during testing is also a typical cause of failure, as grasping *Q*-values remain low. For instance, the robot pushes when it should grasp, and vice versa. 

In [71], their method involves mapping visual observations to two action-value tables in a Q-learning framework utilizing fully convolutional action-value functions (FCAVFs). These two value tables deduce the usefulness of pushing and grasping actions, with the highest value corresponding to the ideal position and orientation for the end effector. To facilitate grasping, they introduced an active pushing mechanism based on a novel metric called Dispersion Degree, which measures the degree to which objects are spread out in their environment. They used two FCAVFs for each grasp and push to be trained in a parallel manner. This means that the grasping and pushing *Q*-values are individually generated to be later selected for each action desired to be performed. The behavior of the push action, however, acts to detach the objects’ arrangement by pushing the entire pile. Even though their approach can separate the objects from each other, it takes several iterations to complete the task, just as in [68,69]. This challenge of push behavior remains unsolved in their approach. The drawback of their approach, which could cause difficulty during grasping tasks, is the coordinating mechanism based on Dispersion Degree, which was used in their work. This mechanism works once the objects are well-organized and very close to each other, because they have calculated the dispersion degree of cluttered objects as a whole pile, such as the objects’ arrangement in the Figure 4b. In addition, once the robot performs a pushing action and the objects remain close to each other, their mechanisms will definitely execute the push even though the grasp can be performed instead.

Alternatively, a depth-by-poking method was proposed to estimate depth from the RGB-D images by using labels from the physical interactions between the robot and its surroundings [72]. Their network calculates the z-plane that a robot’s end-effector would reach if it attempted to grasp or poke the precise pixel in the input image. Then, the deep FCN converts the RGB or noisy depth images into correct depth maps. In [70], the multi-view and change observation-based approach (MV-COBA) was proposed to synergize the push and grasp actions using deep Q-learning.

Notably, the suction grasp requires a more precise and robust grasp point, which necessitates additional data gathering. Furthermore, in situations where the object is partially covered by other items, a feasible grasp pose might not be available, leading to grasp failure. In addition, the surface of the suction material and the surface of the object can induce inefficient poking. A multifunctional gripper (i.e., the use of suction and fingers at the same time) would have been much more effective in such scenarios. Synergizing non-prehensile and prehensile actions (e.g., push-to-grasp, shift-to-grasp, and poke-to-grasp) was found to be effective in completing object removal tasks in cluttered environments, particularly in situations where objects are tightly arranged [73]. Even though the reviewed papers presented sophisticated techniques to address the different challenges, this domain still needs further investigation. 

Recently, a multi-fingered push-grasping policy was proposed using deep Q-learning that creates enough space for the fingers to wrap around an object to perform a stable power grasp using a single primitive action [74]. A target-oriented robotic push-grasping system was proposed that is able to actively discover and pick up the impurities in dense environments with the synergies between pushing and grasping actions. In their study, Target-Centric Dispersion Degree (TCDD) was introduced to perform an active pushing mechanism where the targets are isolated from the surrounding objects [75]. A Deep D-learning-based pushing–grasping collaboration was presented. In their study, they employed two cameras to observe the robot’s workspace from dual viewpoints. Then, they trained the Q-learning on two FCNs, where each FCN received RGB-D data from both cameras [76]. Push-to-See was introduced to learn Non-Prehensile Manipulation for Enhancing Instance Segmentation via Deep Q-Learning [77].

In summary, there are some difficulties that arise as a result of combining two actions. From the literature, in object removal tasks, the pushing action is executed alongside the grasping action by using the explore probability via the max prediction. The pushing and grasping actions were synergized to support two parallel FCNs mutually for grasping, where the second is used for the push. As a result, this strategy of synergizing fails in certain cases because the robot proceeded to push the entire pile of objects, causing the items to be pushed out of the robot’s workspace. In addition, it performs a push movement when it is not necessary, resulting in a series of grasping and pushing actions due to the estimating of the grasp point and push direction on the separated FCN. Another challenge dealing with the pushing behavior is that the push action could be less efficient when it is used in random clutters, where many objects will tend to be pushed out of the workspace. The reason for this issue is that the best action for the robot to take is determined by maximizing the probability of a heatmap. Then, the robot will identify all of the clutter and presumes push action as the best action. As a consequence, it necessitates extensive training iterations, which is time consuming, in addition to more robust training to overcome the challenge of active affordance prediction.

Synergizing non-prehensile and prehensile actions (e.g., push-to-grasp, shift-to-grasp, and poke-to-grasp) was found to be effective in completing object removal tasks in cluttered environments, particularly in situations where objects are tightly arranged. Even though the reviewed papers have presented sophisticated techniques to address the different challenges, this domain still needs further investigation. Table 3 summarizes the related research materials and their item-based comparisons.

### 5.2. Assembly and Rearrangement Task

The assembly task entails the process of assembling several objects in a variety of different shapes (e.g., building a tower or toy block shapes). While the arrangement task is similar to the assembly task in principle, it is focused on the arranging of objects in a basic way (e.g., sorting objects based on color, sorting objects in a line manner). Assembly and rearrangement are robotic tasks that use grasping to complete a specified task in a clutter challenge (e.g., stacking objects on top of one another to create a tower or reassembling the shape of kit toys). The assembly and rearrangement tasks are examined and analyzed in this section to give a clear analysis of the approaches that have been used to address these kinds of tasks.

#### 5.2.1. Assembly Task

The Amazon robotic picking challenge has made significant progress in picking objects from a cluttered scene to achieve assembly tasks, but object placement remains a challenge. Prior studies have neglected to address the placement challenge in addition to the grasping challenge. This section discusses the studies that have been devoted to the actions of grasping and placing objects into assembly tasks. Overall, learning algorithms and detecting object models remain challenging tasks and thus need further investigation.

In [79], an approach was proposed to tackle a difficult class of pick–place and re-grasping issues, in which the precise geometry of the objects to be grasped was uncertain. The motivation of their work is to learn robot grasping and the placing of objects with no prior geometric knowledge of the target object. The researchers utilized a variant of Sarsa and collected n-episodes of experience rather than executing a single stochastic gradient descent step after each event, which is comparable to normal DQN. The system in their scheme learnt 6-DoF pick-and-place actions via simulation, and their findings were applied to real-world cluttered scenes. However, the robot’s capability to generalize unknown object classes and learn new geographical locations was limited. The authors also defined the problem of “reach actions” in their perspective, and the set of target positions that could be reached using the actions was sampled at each time step. Their approach could train each class independently. However, as only two types of object classes (e.g., mugs and bottles) were considered, their findings do not appear to be generalizable to most object classes. Furthermore, as their work considered “distracting” information created by other objects near the target object, their approach performed poorly when training on a single scenario and evaluated in a cluttered environment. The issue of distracting information from neighboring objects can be alleviated by employing the segmentation method.

In another work [50], the researchers proposed a method based on the combined learning of instance and semantic segmentation for robotic pick-and-place with high occlusions in a cluttered environment. Learning the occluded region segmentation with CNN-based pixelwise score regression and learning the combined instance and semantic segmentation, such as visible and occluded regions, are two of their main contributions. This segmentation could be used for a variety of pick-and-place tasks, such as identifying fully visible objects for random picking and picking up obscured target objects. The instance and the image-level reasoning of mask prediction were combined on several segmentation tasks in their work, an action that was lacking in prior studies that only aimed to learn instance segmentation. Berscheid et al. [80] trained a robot to pick and place objects via self-supervised learning without considering an object model. They combined robot learning of primitives estimated by FCNs and one-shot imitation learning (IL). They defined the place reward as a contrastive loss between real-world measurements and task-specific noise distribution to execute the “precise pick-and-place without object model.” They provided a method for effectively handling the Cartesian product of both grasping and placing action spaces $\left[A_{g},\;A_{p}\right]$.

Su et al. [81] proposed an active manipulation of two cooperative robotic arms to address the issue of object occlusions amid the clutter. Initially, semantic labels were generated with affordance prediction based on brand-name and active manipulation (e.g., two cooperative arms and grippers). A vacuum gripper was used to pick a target object based on the affordance prediction at the object or brand-name level. The brand name was then utilized to estimate the grasping, and a two-finger gripper was employed to place the target object on the shelf. Although the abundance of virtual data and their dynamically annotated labels could ensure scalability to a large number of products in real-world shops, many of the automatically produced brand names may be too tiny or occluded to be acceptable for model training convergence. The proposed virtual datasets should be incorporated in future studies to improve performance. Training sets with numerous labeled objects may be constructed in cluttered environments, and this approach can also improve the affordance and grasp predictions.

Object manipulation necessitates planning in a continuous space, which is not achievable in existing hierarchical POMDPs. Further dividing the task is much more challenging. Small POMDPs that depend on the subsets of the entire state and action spaces are also ineffective. Furthermore, not all actions can be implemented whilst manipulating objects. Only effective motion planning allows the actions to be executed. Online POMDP planning searches for a belief tree with multiple action branches, and it uses a wide action space for object manipulation. Motion planning must be performed multiple times because the feasibility of each action branch needs to be assessed. A hierarchical POMDP planning process was proposed in [82] for an object-fetching task that employed a robot arm. A hierarchical belief tree search technique can be used to ensure efficient online planning. This scheme suggests that many fewer belief nodes can be produced by utilizing abstract POMDPs to create part of the tree, and motion planning can be invoked fewer times by determining the action feasibility using the abstract POMDP’s observation function. In [82], an abstract POMDP was constructed manually using domain knowledge, and the performance was affected by how the information was extracted. In the future, as a further expansion of this work, automatic extraction of abstract POMDPs may be considered.

It is currently difficult to handle long-horizon challenges by RL algorithms, and time seems to be wasted studying negative cycles and task progression that can be swiftly reversed. Learning multistep robotic tasks in the real world is extremely difficult. The ability to perceive the immediate physical impacts of an action on the overall progress in handling an object should be prioritized. For example, a schedule of a positive task framework was proposed in [83]. It incorporated common-sense constraints in a way that could significantly enhance both learning and final task efficiencies. The approach architecture in [80] was substantially identical to that in [65] in terms of the notion of combining grasping and pushing, but the researchers in [80] also expanded the topic to include the place action for the stacking objects task. However, the RL approach in [80] required a significant number of time-exploring behaviours, which was somewhat inefficient. In reference [84], the authors attempted to solve the problem of stacking multiple blocks into a tower by using an increasing number of blocks. They proposed a basic curriculum method in which the number of blocks increases when the agent learns a goal task with fewer blocks. The curriculum was supplemented by the attention-based graph neural network (GNN), which provided the necessary inductive bias for transferring knowledge between tasks with varying numbers of objects. They also used the attention-based GNN to train a policy and subsequently address the problems of curriculum learning in multi-object manipulation tasks. They proposed a basic but successful RL-based approach for stacking blocks, with few assumptions regarding the task structure or the environment. Furthermore, object-centric representations were used in their robotic manipulation method, starting from the pick-and-place action for the stacking block task (a task-specific data gathering process) and extending to undetectable objects with substantial shape variations. Although their method was based on the GNN to represent the relationships between objects in a scene, this method frequently relied on a pre-set number of objects.

#### 5.2.2. Rearrangement Task

The task of rearranging multiple objects is no less important than stacking objects, such as towers. For instance, an iterative local search (ILS) entailing heuristics and an ϵ- greedy scheme was implemented for non-prehensile rearrangement in [85,86]. The authors claimed that ILS was equipped with strong heuristics and an ϵ- greedy rollout policy for successfully solving various tasks for tabletop rearrangement, including task sorting. Both [85,86] employed the Monte Carlo tree search (MCTS) equipped with a task-specific heuristic function. A similar work has been reported in [87]. However, the addressed sorting problem in [87] differs from that in [85,86] in two crucial ways. Firstly, in [85,86], specific target locations for each class are supplied as the input for the sorting objective, which eases the task of detecting the misplaced objects and their target locations. In [87], the target destinations are not pre-defined; instead, the planner determines acceptable places to create a sorted state on its own. Secondly, the manipulators used in [85,86] can move the pusher in and out of the pushing plane at any position, while the motion of the pusher used in [87] is restricted to the pushing plane, forcing it to navigate around objects and obstacles.

### 5.3. Object Retrieval and Singulation Task

Currently, retrieving the target object from its surroundings in a cluttered environment is a challenge. Moreover, the object singulation task is no less challenging than the object retrieval task. Both are considered to be the same challenge as they work in an integrated manner. In this part, we review the research studies that focused on addressing these problems by proposing several approaches.

#### 5.3.1. Object Retrieval Task

One type of cognition task is known as an object retrieval task, and it entails finding and grasping a particular object from its environment. Over the past five years, researchers have dug deep into the problem of retrieving an object from a cluttered environment or finding an unseen and obscured object amidst a cluttered pile. All these studies are focused on one singular goal: determining how to find an object, whether it is visible or not, in a cluttered environment. Several tasks that require the robot to interact with a specific target have benefited from robotic manipulation systems that incorporate vision-based learning algorithms. What if the piles cover the intended target? It is still difficult to find the target and isolate it from its surroundings.

Using deep RL, an autonomous method was employed in [88] to find unseen objects within the clutter. Four RL agents were used to train a CNN using the tabular RL technique. Semantically segmenting the target object allowed the RL agent to learn how to distinguish it from its surroundings. However, their method was constrained by the number of objects that had to be detached in order to retrieve the target, making it less reliable and simple in situations where objects are densely cluttered. It also required more training repetitions (about 20,000 iterations). Chen et al. [89] tried to grasp an object when the object was in three different conditions: fully visible, slightly occluded, and completely occluded. In order to implement such scenarios, they proposed a value-based deep RL that makes use of mask R-CNN to train the grasping policy. The method was trained using a pair of DQNs, one for pushing and one for grasping. In their approach, the synergy problem between pushing and grasping to remove an object from crowded surroundings was solved with the help of the mask R-CNN algorithm, which is used to detect and mask the target objects.

Additionally, Novkovic et al. [90] proposed an interactive perception method for object recognition in clutter based on an RL-based control algorithm and a color detector. Proximal policy optimization (PPO) was used in [90] to predict how an agent will act in the future based on both what it knows from its past experiences and what it knows about its current state. The authors used a robotic arm with an RGB-D camera attached to its wrist to observe it from different angles to ease the grasping of an occluded object. For RL-based object tracking, the scene state was encoded using a discretized truncated signed distance field (TSDF) volumetric representation. Since it might be difficult and intuitive to distinguish between actions that are desirable or undesirable, supervised approaches are often irrelevant for this task. Yang et al. [91] proposed deep Q-learning to grasp invisible objects, which involves two stages. One is to check the visibility of the target objects. For example, if the target object can be seen, the robot will move to the second stage by performing either a push or a grasp. If not, the robot will first explore the target object by continuing to push until the target object is found, and then the robot will go through the coordination of grasp–push actions to grasp it. In contrast to Yang et al. [91], another study [92] proposed a graph-based deep reinforcement learning model to effectively explore invisible objects and enhance cooperative grasping and pushing task performance.

The work in [90] discretized representation to encode observation history, affecting next iteration assumptions due to the requirement to “forget” volumes where objects moved. Moreover, some end-effector positions are kinematically impossible in real life, and test scenarios had less clutter. Similarly, the pushing reward function in [91] was constructed manually; it may need several tuning trials and lack adaptability. Furthermore, the authors in works [90,91,92] assumed prior knowledge and relied on a target object with a specified color to be retrieved. In contrast, Fujita et al. [93] accepted the target object as an image instead of a segmentation module [91,92]. A deep RL system based on active vision has been used to retrieve in dense clutter. The QT-Opt algorithm [38] was adopted with hindsight experience replay (HER) [94] for the goal-conditioned situation. In [95], deep RL was used to train an agent to make continuous push actions that help the gripper clear away clutter or push the target object out of the clutter for retrieval when it is hidden by a pile of unknown objects. However, due to a number of failure factors, the agent could not always fully uncover the target object or enhance grasping. For example, the agent could have repeated executing the same action over and over again without changing the environment, or it could have moved close to the target object without interacting with the objects that occluded the target object.

Another study [96] developed a framework for robot learning, which is called “Learning-guided Monte Carlo tree search for Object Retrieval.” In order for a deep neural network (DNN) to comprehend the complicated interactions between a robot arm and a complex scene with many objects, Monte Carlo tree search (MCTS) is initially used. This allows the DNN to partly clone the behavior of MCTS. The trained DNN is then incorporated into MCTS to direct its search process. Additionally, the study in [97] has proposed a framework for learning to train a scene exploration strategy that is efficient at finding hidden objects with little interaction. To begin with, scene grammar was described as organized clutter. Then, using deep RL, a graph neural network (GNN) was trained as a based Scene Generation agent to manipulate this Scene Grammar and produce a variety of stable scenes, each including several hidden objects. Deep RL was used to teach a scene exploration agent how to find hidden objects in these kinds of crowded scenes.

The X-ray mechanical search approach was developed by Danielczuk et al. [98]. It divides the process into “perceiving” and “searching” for the optimal grasp. The RGB-D data was sent into a network to be used in the perception phase. The bounding box of the target object and the augmented depth image were utilized by the network to estimate the occupancy distribution and increase the grasp success rate for occluded objects. During the perception stage, a set of segmentation masks were also constructed. In the searching stage, the X-ray mechanical search strategy recognized the mask on the target object and prepared a grasp on that mask. Deng et al. provided a description of an alternative method to retrieve a target object based on the question-and-answer (QA) policy using the manipulation question-answering (MQA) method, in which the robot would employ manipulation actions to change the environment in order to answer a question. Deng et al. [99] have a QA module and a manipulation module in their framework. In the QA module, the visual question-answering task is used. In the manipulation module, a DQN model was also used so that the robot could use manipulation to interact with its environment. They explored a situation in which a robot might manipulate objects inside a bin until they received the answer. Different sorts of questions in the dataset are reasonable from a research perspective, and the MQA system’s performance should be enhanced.

A goal-conditioned hierarchical RL formulation with a high sample efficiency was given in [100] to train a push-to-grasp technique for grasping a specific object in clutter. In [101], the main objective was to use quasi-static push and overhand grasp movements to extract a target object from a densely packed environment. The visual foresight tree (VFT) method was presented to identify the shortest sequence of actions. The method combined a deep interactive prediction network (DIPN) for estimating the push action outcomes, and the Monte Carlo tree search (MCTS) for selection of the best action. However, the time required by VFT is long due to the large MCTS tree that must be computed. Moreover, it assumes prior knowledge and relies on a target object with a specific color. These studies concentrated on object retrieval task rather than the objects removal task. They assume prior knowledge and rely on a target object with a specific color.

Applying a mask of the target object to the grasping map is necessary to extend these techniques to goal-oriented tasks; this is the same as the idea behind the approach in [91]. However, the approach in [91] chooses the actions for grasping the undetected object using a classifier, which significantly relies on the accuracy of depth data. This concept could provide an explanation for why their policies regularly fail to decide when to grasp. Additionally, the task took a long time to complete for the VFT restrictions in [101] because of the enormous MCTS tree that needed to be computed. It would also be interesting to construct a network for directly analyzing the reward for the distribution strategy, which is useful in determining the cost of implementation, even if MCTS can now be performed with only one thread. Their approach is limited to one color and cannot be used to retrieve an object of a different color without first being trained on that color.

Furthermore, if there are two blue objects in the scene, would their technique be able to collect both of them, or would it just be sufficient to retrieve one and begin a new trial? A hybrid planner based on a learning heuristic was employed by Bejjani et al. [102] to address the issue of occlusion for lateral access to shelves. They demonstrated how to use a data-driven approach to build closed-loop systems that are aware of occlusions. The hybrid planner was used to look at different states that came from a learning distribution across the target object’s location. Even though their technique could find different objects, they used the Alvar (AR) tag tracking library to recognize object positions and clutter. As seen in their demonstration video, their approach only works with AR-tagged target objects, and the tag must cover all of the object’s faces. The question is, “Will the AR-tag work if it is attached to one face that is not visible to the camera?” Moreover, object retrieval from a confined environment has been addressed using the DQN-based Obstacle Rearrangement (DORE) algorithm as proposed in [103], and the success rates were checked using intermediate performance tests (IPTs). In the event of a failure, the algorithm employs a transfer learning technique. The features of actions were used to construct two networks: a single DQN and a sequentially separated DQN. Table 3 highlights the most important studies that attempted to address the problem of retrieving objects in a cluttered environment.

In conclusion, the retrieval object task has been the focus of a significant amount of research yet continues to be challenging. Even though the retrieval object task has been extensively researched, some difficulties will need to be resolved in order to enhance the retrieval object pipeline in the future. Table 4 highlights the most important studies that attempted to address the problem of retrieving objects in a cluttered environment.

#### 5.3.2. Singulation Task

A singulation object is a task that involves isolating an object from its crowded surroundings such that the robot may quickly reach the target. The receding horizon planner (RHP) was proposed in [104] to retrieve the target object via pushing manipulation in a clutter situation. The authors of the work explored how to identify a suitable function-based heuristic for planning and estimating the cost-to-go from the horizon to the target. The DQN method was trained on a dynamic neural network to decide the executable actions. They also proposed a RL policy in the form of an RHP to choose a random pushing action with a chance of the policy querying the RHP for the movement. Addressing the challenge of reaching the target in the clutter is difficult, as it requires rapid planning and reliable performance in the manipulation task. To enhance the pushing behavior, the study in [105] proposed the combination of image-based learning systems with look-ahead planning. The authors intended to use object geometries to improve the efficiency of manipulation movements. However, this approach generally necessitates knowledge of the position and shape of obstacles. In reference [106], the work’s contributions were built on the research on heuristic learning for RHP in a discrete action space [104] and learning transferable manipulation skills through abstract state representation [105]. The researchers of the work extended the contributions to gain manipulation actions in a continuous action space, reducing by threefold the number of required pushing movements to solve a manipulation problem compared with a discrete action space. In [105], two heuristic learning approaches were used in discrete and continuous action spaces. The researchers advocated modifications to the existing IL and RL methods as a means of improving the learning algorithm’s stability in sparse-reward environments with nonlinear and non-continuous dynamics.

Furthermore, despite the complexity of a scene, no mutual support nor occlusion exists between them [107]. The approaches in [96,97,99] have different degrees of success; nevertheless, due to the high-dimensional and under-actuated issues and the uncertainty of real-world physics, the complex scenarios involved in this problem remain challenging for autonomous systems. To mitigate these issues, approaches utilizing randomized planning have been proposed. Papallas et al. [108] proposed human guidance to solve the issue of reaching through a clutter. The approach required human inputs to be fed before planning. It also used a sampling-based planning approach as opposed to the trajectory optimization approach. However, trajectory optimization is more appropriate for online replanning as it entails warm starting the optimization with the trajectory from the previous iteration. In [109], Papallas et al. subsequently proposed the use of trajectory optimization with human input. Their techniques are only concentrated on isolating the target object from its surroundings. However, they do not consider the surrounding objects while attempting to reach the target object. In this instance, the value function is learned from predefined features, which limits the framework’s applicability to certain single-shaped objects, as described in [96,98,99,100]. In addition, those methods presupposed a predefined collection of geometric descriptions of real-world objects and used Cartesian coordinates to express the state. In simulated and real tests, these methods are limited to singulating, separating, organizing, and sorting large-scale clutters. Randomized planning is one of the most recent techniques of clutter manipulation. However, the issue remains challenging because planning timeframes are still in the tens of seconds or minutes, and success rates are low for difficult situations.

Grasping of objects in a cluttered environment is challenging because the target object might be laid closely with the surrounding clutter, resulting in a lack of collision-free grasp affordances. A failed grasp occurs when the target object is either touched or occluded by other items in the scene. The robot is unable to execute collision-free grasping due to the object’s closeness to the box borders or other items. When an object is pushed, it is also isolated, allowing the manipulator’s fingers to be positioned. In [110], the problem of object singulation in a cluttered environment was addressed to help grasping. The authors of the work proposed lateral pushing movements to separate the object from its surrounding clutter, a scheme comprising previously undetectable objects by using the fewest number of achievable pushes. They leveraged RL to determine the optimal push policies based on in-depth observations of the scene. The action value function could be estimated using a deep neural network (Q-function). Although a high singulation success rate was obtained in the simulation, the singulation is inappropriate in real-world settings because of the slow convergence of the network. In addition, the approach has limitations in terms of barrier height assumptions. Split DQN [111] can offer a solution to this problem by demonstrating faster convergence to the optimal policy and offering a generalization to the additional scenes. Although [111] only employed a single policy (max-value between the *Q*-values of the discrete directions), it could determine several policies, namely, basic policies and a high-level policy. Both [110,111] focus on teaching a robot to isolate objects from its surroundings by using a push action, but the method’s performance with more primitive actions, such as pick-and-place action, has not been evaluated. Training the robot in even more difficult circumstances, such as scenes where the target object is blocked by other items from the top, remains a problem. Both methods may be extended by determining whether continuous actions can produce optimum policies for this sort of complex situation.

In contrast to the work in [110,111] that utilized discrete actions to isolate the target object, the work in [112] used continuous actions to provide the agent with additional options for pushing. The work proposed a modular RL technique to entirely isolate the target object from its surroundings by using continuous actions. The heightmaps of the scene and the mask of the target were utilized to create the visual state representations. An autoencoder (AE) was used to reduce the dimensionality of each observation state. Then, the AE’s latent vectors were sent for high-level policy, which then determined which primitive policy to use. As a result, a high-level policy was used for selecting amongst several pushing primitives that had been trained individually. With the action primitives and feature selection, prior knowledge could be efficiently incorporated into learning, thus boosting sample efficiency. However, even though the approach is efficient at isolating a target object in a crowded environment, it is only intended to isolate the object without grasping it. This aspect should be examined in the future, coupled with the grasp action, to not only isolate but also pick up objects in cluttered environments. In addition, for future work, this technique may be useful in the assessment of the policy for non-convex geometry objects.

The current approaches are limited because they either model systems with a fixed number of objects or use image-based representations whose outputs are not interpretable and quickly accumulate errors. In reference [113], a GNN-based framework was proposed for effect prediction and parameter estimation of push actions by modeling object relations based on contacts or articulations. However, as reported, this method relied on a predefined number of objects. Furthermore, it only considered simple scenes, such as falling blocks, and did not involve applied actions for robotic manipulation tasks. The framework is generic and might benefit from intelligent exploration strategies in order to be generalized to a changing number of objects. In addition, the learning of unsupervised representations for objects via interactions is a powerful tool for the visual grounding of objects. In another work, Won et al. [114] proposed a method to produce nonlinear pushing movements for object singulation based on an off-the-shelf machine learning algorithm and a conventional semantic segmentation process. Whilst dexterous grasping for various shapes of objects was not considered, they focused on mitigating the clutter near the target object.

## 6. Challenges and Future Directions

In the last six years, robotic grasping has been extensively investigated, and several approaches have been dedicated to addressing many challenges in a cluttered environment. Although grasping of objects amid cluttering is a trivial task for humans, it is challenging for robots. This section highlights a few more challenges and the potential future directions. The discussions are expected to help researchers who are interested in working in this domain.

### 6.1. The Challenge of Sole-Grasping Policy

Recent studies that focused on multi-finger grasps (e.g., [37,39,47]) primarily employed planned precision grasping for known objects. These types of approaches require complete knowledge of the object’s position, mass, material, and shape to achieve a final grasp. As a result, reliance upon that particular modeling renders grasping planning prone to inaccuracy with unknown objects in real-world situations. Furthermore, a mapping between the hand attitude and the local geometry of graspable objects may be learned by evaluating the poses of successful grasps over a large dataset. As proposed in [115], the geometric approach to multi-finger grasping takes shape complementarity between the robot hand and the target into account. However, grasping in crowded settings with a multi-finger gripper is inefficient, particularly when dealing with tiny objects or retrieving a target object from the clutter, since the multi-finger hands may struggle to find enough space for their fingers to move freely in the workspace. In comparison, other studies have concentrated on robotic grasping of unknown objects adopting parallel-jaw grippers (e.g., [30,36,41,43]). These studies use a noisy and partially occluded point cloud as input and generate pose estimations for feasible grasps as output. However, template matching cannot perfectly deal with self-occlusion and mutual occlusion between objects. Moreover, batch-training might not be ideal for predictions dealing with heavy clutters. As stated in [116,117], multi-finger grasps remain more problematic than parallel-jaw grasps.

Multifunctional gripper-based grasping is a valuable ability for grasping in a cluttered environment whilst keeping an affordance-based grasp position detection. This mechanism has been trained with self-supervised learning and deep RL in several studies (e.g., [61]). The majority of them attempted to efficiently accomplish the cleaning objects task. Even though they were able to effectively grasp the object, certain challenges must be overcome for the mechanism to be generalized in other scenarios. Suction grasping, for example, recorded failures when the grasp point was estimated on the edge of the object, causing the object to slide before it was placed [61]. It was also found to be less efficient when dealing with deformable objects because of poor shape prediction [58]. To ensure learning convergence, deep learning models still require a large volume of labeled data. Researchers must seek ways to learn and estimate object affordances with fewer manually annotated samples.

The existing state-of-the-art GCN-based techniques mostly focus on the gripper end-effector. In [118], a GCN-based method was proposed to predict object affordances for grippers and suction end-effectors in bin-picking situations. To overcome the existing generalization and scalability constraints, the grasping approach was extended to 6-DoF in a more flexible manner. Specifically, the proposed suction approach fully used the 3D environment, and grasps were predicted and executed with 6-DoF pose estimation of objects. In contrast, the grasp affordance with the gripper approach was predicted using vertical grasps and discrete angles. As a result, the computational cost was proportional to the selected number of discrete angles, making the solution difficult to scale. Additionally, the use of CNNs can substantially enhance grasping accuracy; however, the annotation of the grasping point is costly (e.g., [60]). Contrary to conventional image classification algorithms (e.g., CNNs), the weighted ensemble neural network [119] can effectively overcome the difficulty of uneven placement and illumination across an image, which can affect the grasp point estimation during learning. D’Avella et al. [120] created a collaborative robotic system that combined a conventional two-finger gripper with a low-cost custom universal jamming gripper (UJG). However, it is expected to be more efficient if the same concept can be trained using self-supervised learning (deep-LR). In the future, combining perception algorithms with UJGs might be useful in industrial applications such as bin picking. In conclusion, the difficulties associated with removing objects that use the sole-grasping mechanism continue to be a source of concern and demand additional investigation.

### 6.2. The Challenge of Synergizing Two Actions

Synergizing two actions is another mechanism for addressing the challenges that sole-grasping and multifunctional grasping policies cannot handle. The difficulty in a situation involving well-organized objects is that there is no space for the robot’s gripper’s fingers to execute the grasp. To overcome this challenge, previous studies have focused on synergizing push and grasp using the max-value strategy (e.g., [62,65]), which is trained on 2NNs, one for each action. The issue continues as a result of an inefficient push: (1) favoring push over grasp, which results in the entire pile being pushed out of the workplace; (2) the robot might conduct several pushes to detach the objects from their arrangement; and (3) the robot performs a push when the grasp action should be performed instead. Furthermore, because of push behavior and the workspace size, synergizing grasp and push would show limited performance when it is applied in random crammed clutter scenarios. The reason is that the max-value strategy clearly prioritizes pushing over grasping. As a consequence, the robot continues to randomly push amid a cluster of objects, thereby pushing them out of the robot’s workspace. Additionally, many iterations will be conducted to complete the task; in some cases, the robot may fail to complete the task successfully because of the many items being pushed out of the workspace.

To prevent pushing the entire pile, certain studies advocated shifting objects [67] (e.g., putting the gripper’s finger on top of the object and then performing a shifting) rather than pushing them. Alternatively, rule-based approaches (e.g., [66,69,71]) have been proposed to address the problem of avoiding excessive push, but they still rely on the max-value policy. The problem still exists since pushing and grasping rely on the max-value, which is learned using independent NNs. A variety of approaches could be considered in order to address this issue. The first recommendation is to use the explore-to-coordinate concept, which involves segmenting objects and masking the target that has to be grasped or pushed during the explore stage. Then, as in [91,100], evaluating the situation in order to coordinate a grasp or push action could be another alternative. The policy (e.g., [91,100]) was used to retrieve the target object, but it could also be applicable for clearing objects instead of retrieving them. Furthermore, rather than utilizing a parallel-jaw gripper to push or grasp, a multifunctional gripper (combining a parallel-jaw and suction as in [61]) may improve the effectiveness of the learning grasp in a cluttered environment. For instance, pushing or shifting the item may be accomplished with a parallel-jaw finger, while grasping could be accomplished by coordinating the parallel-jaw finger and suction mechanism. This strategy is expected to minimize grasp failure because the multifunctional gripper may push and grasp simultaneously by coordinating the finger and suction mechanism.

### 6.3. The Challenge of Assembly and Rearrangement of Objects

In a block-stacking task, humans know that grabbing at empty air would never catch an object, but a deep RL algorithm may take some time to figure this out. For example, [83] completed the task of having a long horizon (e.g., stacking objects on top of one another like a tower); however, it is highly demanding in terms of data and requires several iterations to be more efficient in implementing such a task. As a result, it should leverage reactivity and failure recovery to compensate for the loss of precision caused by the policies trained in simulation and in the real world. Learning the long-horizon tasks remains a challenge because current studies (e.g., [83,84]) have focused on limited learning tasks that may not be generalizable in another scenario with a diverse set of objects. Thus, learning the long-horizon tasks for lifelong learning, in which the technique can gather a large number of instances autonomously, could be a future path to be addressed.

One of the challenges is that learning of robotic manipulation tasks using RL with sparse rewards is presently unfeasible due to the enormous amount of training data needed. For example, many practical block-stacking tasks involve the manipulation of multiple objects, and the difficulty of such tasks increases as the number of objects increases. Although learning from a curriculum of increasingly difficult long-horizon tasks appears to be a natural solution, it does not work in many situations [84]. As pointed out by [121], such approaches include only simple situations, such as falling blocks, and do not contain behaviours relevant to robotic manipulation tasks. Currently, the curriculum is being produced manually and is based on the premise that smaller groupings of objects have easier learning and control capabilities than larger groups of objects. However, more complicated and effective curricula may exist along axes of variation other than object cardinality, and identifying these curricula would be an exciting area of future study. Learning the long-horizon tasks is not less difficult than learning the task of rearranging multiple objects (e.g., [85,86,87]). Calculation time is quadratic in terms of the number of objects in such methods, which is problematic. The development of computationally efficient algorithms is required to ensure the large-scale viability of these approaches in situations involving a large number of objects. It is also recommended to include visual and other sensory observational investigations.

### 6.4. The Challenge of Object Retrieval and Singulation Task

The object singulation task is a method of separating items from their surroundings so that they can be later retrieved. Singulation is considered to be the pre-movement that can be performed by a robot before retrieving the target objects. Due to the fact that the singulation task and the retrieval task share the same challenge, we focus on the retrieval task in this section. Thus, the method that has been suggested to complete the task differs, making the retrieval task challenges parallel to the singulation task challenge.

Multiple studies emphasizing the segmentation performance-based retrieval of a target object in cluttered environments have been published recently. The focus is on retrieving objects of a certain color that have previously been known. This type of approach can cause a variety of issues. For example, in [90,91,95,98], to be able to grasp the target, the target object should have a specific color; otherwise, it will fail to find the target. The question “are their approaches capable of grasping the target object that is not contained in the priory specified colored-target objects?” remains unanswered. In a scenario with two target objects of the same color, another problem is “will their approach retrieve both or will it suffice to grasp one and start a new test?” Other studies have also proposed to incorporate barcodes or quick response codes (e.g., [102]), which are affixed to the object to be detected. This was restricted to tags on the target object, which must cover all of the object’s faces. The concern is “will the VAR-tag work if it is attached to one face and that face is not visible to the camera?” This remains a difficult domain, since their approaches assume prior knowledge and rely on a predefined target object either using color or tag-free (e.g., QR code and barcode). It is recommended to be thoroughly studied to resolve these constraints. Lastly, the exploitation of embodiment is a technique for leveraging language-based grasping learning. This applied strategy also allows for the smooth integration of language learning with RL, as presented in [99], by allowing the varied tasks (e.g., object retrieval task or clearing object task) to be executed. In embodied language learning in a dynamic world, deep RL neural architectures offer a potential research path for the processing of many modalities. We refer the interested reader to the latest survey in [122] for additional information in this domain.

### 6.5. The Challenge of Grasping Deformable, Transparent, Black and Shiny Objects

Detecting deformable, transparent, black, or shiny objects amid clutter remains difficult, and it has not been studied as much as the other types of objects. Imagine a cluttered scenario consisting of mixed transparent, deformable, shiny, and black objects. A possible concern is “can the robotic vision detect these objects as it has been with the other types of objects?” Transparent objects are a frequent sight in everyday life, but they have unique visual characteristics that make accurate depth estimations with ordinary 3D sensors extremely difficult. The work of [123], for example, is a recent effort that emphasizes this difficulty. They introduced the ClearGrasp concept and used deep learning with synthetic training data to infer the correct 3D geometry of transparent objects from a single RGB-D image. However, their technique posed a serious problem; that is, they used a pre-trained model that is not always effective in all circumstances, resulting in point cloud noise that can impact the prediction grasp point. As a potential future path, incorporating vision-guided exploration with RL-based action model training (e.g., [38]) might be a viable strategy in situations where data efficiency is the learning constraint. Alternatively, several works have concentrated on deformable objects [124] or rigid and soft objects that are randomly piled together [125]. In [125], even though the method was only tested on a few types of deformable objects, it could be extended to incorporate an RL framework in the future, which we believe can enhance grasping in a cluttered environment for the removal or retrieval object tasks. Regarding shiny or black objects, the issue of “is there a method that can grasp these types of objects once they are placed together in cluttered scenarios?” remains unresolved. This area requires further research as a potential direction for academics.

## 7. Recommendations

Some recommendations for future research direction are discussed in this section. We believe it would be valuable for academics to further explore this domain by using the information provided in this survey.

Firstly, it is recommended to achieve comparable object detection and posture estimation quality by simply using simulated data. This approach could reduce the need for access to a robotic setup for adapting the learning method to real-world situations. For instance, it would be interesting to expand the training procedure to allow the semantic segmentation of scenes as implemented in [30,55], which may lead to a more robust posture estimate when considering the model as described in [126]. Additionally, as demonstrated in [127], it would be noteworthy to include a learnable function that incorporates object and contact physics into the prediction model of [31].

In general, view-based rendering is insightful for a variety of activities requiring ego-centric visual states and action spaces. More research is needed to determine the benefits of view-based rendering in other applications (for example, navigation or placement [128]). In terms of convergence, deep learning models still require voluminous labeled data. Researchers must seek ways to learn and estimate object affordances with fewer examples that have been manually annotated. Even though existing state-of-the-art GCN-based techniques focus on the gripper end-effector, a comparison of [118] with other methods in a bin-picking situation would be beneficial. Consequently, the grasping approach needs to be extended more flexibly to 6-DoF configurations to overcome the existing generalization and scalability constraints. Moreover, Q-PointNet was proposed by Wang and Lin [129] to detect a partial object in cluttered space. The RGB image is used as the input of the mask R-CNN to generate a mask from the target object. The depth image and mask images are combined to form a partial point cloud. The partial point cloud is then fed into the PointNet feature extractor. This sort of deep learning model is advantageous and can be used in tandem with the RL framework to enable the robot to either clear or retrieve objects. Secondly, PointNet++ [130], another deep learning structure, could be used instead of PointNet because it outperforms PointNet and is more resistant to point cloud noise.

Secondly, certain failure instances occurred in [47] as a result of the high-level clutter, which reduced the likelihood of the robot putting its fingers. To bridge this gap, it is recommended to combine tactile-enabled BH-282 Barrett hand-based object handling with Multi-Fingered Adaptive Tactile Grasping (e.g., [131]). Alternatively, GenerAL [47] could be well trained to learn the graspable point and pre-grasp finger joint angles, increasing the probability of efficiently grasping. As a consequence, GenerAL may be able to determine which object should be grasped next in a cluttered environment. It is advocated to utilize GenerAL for the aim of grasping a specific object with semantic segmentation methods (e.g., [132]). Another suggestion is to use a separate network for collision detection, as described in [41], to speed up the learning of the avoidance collisions in [48]. Another issue that requires attention is the fact that the high-quality grasps, produced by [48], may not always transfer into efficient grasp prediction in the actual world. For example, instead of directly calculating the quality metric, a critic network can be used to assess multi-finger grasps. As a consideration, the generative grasping convolutional neural network (GG-CNN) [133] seems to be a promising candidate for improving grasping in the presence of clutter. In addition, the work in [49] could be enhanced to incorporate an active perception component, enabling the robot to choose informative perspectives; this strategy can more considerably reduce uncertainty in grasp estimations. Moreover, integrating more sensors (for example, a touch action [134]) and enabling the robot to do additional primitive actions (for example, pushing [65]) are other effective strategies to enhance the resilience of a closed-loop robotic grasping system.

Lastly, according to recent computer vision applications, large manually labeled datasets have remarkable segmentation performance, which necessitates a time-consuming procedure of manually labeling data for new settings. Several robot functions, including grasping, tracking, and object sorting, require visual segmentation of unknown objects. Different works have addressed this challenge, using methods that can be used to alleviate the challenge of finding object in clutters. For example, the transfer learning technique [135] has been implemented by fine-tuning an existing DeepMask instance segmentation network (binary masks). Moreover, a single-stage one-shot shape-based instance segmentation method [136] has been proposed to create the target object’s modal segmentation mask in a depth image of a scene by simply using the target object’s binary shape mask. Even though such approaches entail a particular challenge in the new environment, they can enhance segmentation performance. Instead of looking for a single target object, as in a mechanical search, training on multi-class and multi-object labels can provide a more effective approach for the segmenting situations in grasping. Recently, unsupervised learning methods, such as those presented in [137], have played a significant role in this domain because they offer a direct way of modeling the dynamics of a robot’s interactions from unlabeled 3D point clouds and images. In the future, it will be possible to train structured dynamics models across a wider range of applications by learning to directly infer depth maps from visual observations in a self-supervised manner. The integration of both the inverse and forward dynamics models in the planning is also a potential path for future research.

## 8. Conclusions

Sensing and interpreting the surroundings are fundamental skills for the robot to possess, and assist the robot in performing object grasping in a cluttered environment. Several studies have been devoted to overcoming the difficulties of grasping in clutter, where various tasks have been achieved using alternative perspectives based on deep reinforcement learning. In our review, we divided the task domains into four categories. Some efforts have emphasized achieving the object removal task by picking up all the objects from the robot’s workspace. Others entailed solving the problem of retrieval and singulation of objects from a pile. Several efforts, on the other hand, have been made to execute assembly tasks by rearranging objects in clutter. This review article contributes essential insights about the challenges, future directions, and recommendations by discussing relevant studies on performing grasping tasks amid cluttered environments. According to the key findings, grasping objects in cluttered surroundings remains a challenging task that requires additional investigation to increase grasping performance. We expect that this review paper will help academics to discover knowledge gaps that need to be addressed in the future.

## Figures and Tables

**Table 1:** Essential reinforcement learning terminologies.

Terms	Definition
Reinforcement learning (RL)	A branch of machine learning concerned with how agents should operate in an environment to maximize a notion of cumulative future reward.
Markov decision process (MDP)	MDPs give a general framework for sequential decision making, and the dynamics of an MDP are defined by a probability distribution. An MDP serves as the conceptual framework for RL because it allows the RL interaction process to be expressed in probabilistic terms.
Q-table	A basic table in which the maximum expected future rewards for each state of action are computed.
*Q*-value	The expected return from a given state under a specific policy.
Q function	Once given a state-action pair, a Q function will generate Q values using either state-value functions or state-action value functions.
Value of Action	$\mathrm{The}\;\mathrm{expected}\;\mathrm{rewards}\;Ε\left[R_{t}\right]$$\;\mathrm{received}\;\mathrm{when}\;\mathrm{an}\;\mathrm{action}\;\left[A_{t}=a\right]$ is taken is called the value of action.
Policy *π*	A policy is a manner of describing an agent’s behavior by mapping its current state to a probability distribution over actions π(s|a).
Optimal policy π*	Is one which as good as or better than every other policy. The value function for the optimal policy thus has the greatest value possible in every state.
The deterministic and stochastic policies	The deterministic and stochastic policies are the two most common types of policies observed in the RL domain. A deterministic policy is one that assigns a single action to each state. The action chosen in state *s* by the policy (*π*) is represented by *π*(*s*), e.g., *π*(*s*) = *a*. It means the agent can choose the same action in several states, and some actions may not be available in any state. A stochastic policy is a policy in which multiple actions with a non-zero probability can be chosen. For each state, *π* defines a separate action distribution, e.g., *π*(*s*|*a*).
Exploitation versus Exploration	Exploration permits the agent to have a better knowledge of each action in the long run. The agent can make better decisions in the future by enhancing the accuracy of the estimated action values. Exploitation, on the other hand, takes advantage of the agent’s current estimated values. To maximize its rewards, it selects greedy actions. However, it is possible that by being greedy with estimated values, it will not achieve the best results.
Epsilon-Greedy Action	During exploitation, Epsilon-Greedy picks the action that maximizes the current value estimation, while during exploration, Epsilon-Greedy chooses a uniform action at random. So, the action with the highest value is called a “greedy action,” and the other actions are called “non-greedy actions.”
Discount Factor *γ*	The discount factor basically expresses how important rewards in the far future are to reinforcement learning agents in comparison to rewards in the near future, 0 ≤ *γ* ≤ 1. When *γ* is set to zero, the agent is seen to be short-sighted since the agent is mainly concerned with the immediate reward. When *γ* is close to one, the agent is considered farsighted since future rewards are weighted more heavily than immediate rewards.
Model-Based and Model-Free	In model-based approaches, the RL-agent either has access to the model (environment) and therefore knows the probability distribution across states that the RL-agent ends up in, or the RL-agent attempts to develop a model (often an approximation). In model-free approaches, no model is provided, and RL-agent does not attempt to figure out how it works directly. The data is simply acquired, and then the best policy is developed. Therefore, value and policy iterations are prominent examples of model-based algorithms that estimate the value function with the help of the transition and reward functions (of the provided MDPs).

**Table 2:** Summary of the relevant papers on the sole-grasping policy.

Methodology	Drawbacks	Gripper	Ref.
Deep Q-learning.	Instance segmentation problem in computer vision. Template matching cannot perfectly deal with self-occlusion and mutual occlusion between objects. Batch-training might not be ideal for predictions dealing with heavy clutters.	Parallel-jaw finger	[30]
QT-Opt, an off-policy training method, was proposed on the basis of the continuous action extension of Q-learning.	Learning often requires several robots and devices to compute the vast amount of needed data. In instances where a target object change rapidly (including in the logistics sector), ANN-based grasping methods must be retrained, which is an inefficient approach. High cost of real-world setup with many robots, and high amount of time is required to gain expertise.	Parallel-jaw finger	[38]
The concept of self-supervised learning was applied. The Dense-Object Net, which used ResNet architecture to learn dense visual representations of objects from RGB-D data for robotic grasping in cluttered environments, was introduced.	Only demonstrated a dense descriptor for three different classes of objects, but a larger object class number would hinder the segregation of each class into different parts of the descriptor space. Required a human user to define graspable points in the scene rather than autonomously executing the goal-conditioned grasping.	Parallel-jaw finger	[34]
The pixelwise prediction of multi-class instance masks was incorporated into the mask R-CNN for both visible and occluded region mask segmentation [40]. The combined learning of instance and semantic segmentation was proposed for visible and occluded areas in their next study [50,51]. As a type of pixelwise classification, the semantic segmentation used FCIS architecture to predict position-sensitive masks. For multi-class instance masks, the instance segmentation used the modified mask R-CNN.	Only considered the shapes or geometries of objects, but numerous other factors, such as material characteristics and mass, were neglected; the vision-only technique entailed an open loop and lacked information about object interactions; resilience was difficult to ensure. Required a dataset containing all of the objects’ potential occlusion states and their associated labels and masks; the amount of effort necessary to accomplish this task increases exponentially as the number of objects increases.	Suction grasp multifunctional gripper	[40]
An RL framework was trained on the proposed NN whilst interacting with objects via active learning.	Occasionally failed to grasp objects aligned with the boxed edge.	Parallel-jaw finger	[43]
The basis of the pixel-attentive policy gradient approach, which took a single depth image and gradually zoomed into a particular portion of the image to estimate the optimal grasp.	Two main issues preventing deep learning from being used for multi-finger gripper-based grasping: dependency on external planners to produce alternative grasps due to the wide range of object types and the high dimensionality of multi-finger gripper configurations. the grasping input representations in ANNs increase the search complexity when arranging multi-finger grasps compared with the scheme for parallel-jaw grasp.	Multi-finger gripper	[39]
A real-time, deep convolutional encoder–decoder NN was proposed for open loop robotic grasping by using only the depth image information. By using a depth image, UG-Net predicted the quality and posture of a grasping in pixel-by-pixel manner.	Failed grasps due to the inaccurate z-axis coordinate prediction in the grasp position and an overestimated gripper width that would come in contact with the other parts of the object.	Parallel-jaw finger (dual-arm robot)	[35]
The notion of human demonstration and action-view representations were leveraged by rendering or simulating future states with respect to numerous potential actions. By assessing these states using a learnt value function (e.g., *Q*-value), an end-to-end 6-DoF closed loop grasping model with RL was demonstrated.	Basic view-based rendering was used as a forward-prediction approach; whilst this approach may mimic possible motions and passive observations, it ignored contact mechanics, which was critical for in-contact manipulation.	Parallel-jaw finger	[31]
An RL framework and 3D vision architectures were proposed to obtain feasible gripping viewpoints by using hand-mounted RGB-D cameras. On the basis of their framework architecture, an object detector, a perspective optimizer, and a grasp planner were used to build the modular pipeline. Binary segmentation masks and high-level instructions can be easily comprehended by humans, and they served as interfaces between modules.	Modal segmentation appears to be more successful than the binary segmentation mask employed in this experiment.	Parallel-jaw finger	[36]
ASOR was proposed for the data augmentation approach, allowing for the creation of training data that are suitable for training manipulation in cluttered environments by using demonstrations collected in clutter-free conditions. Two ASOR-based network topologies (e.g., ASOR-IA and ASOR-EA) were constructed, each with its own function.	More complicated manipulation tasks involving numerous objects were included in the immediate extensions.; task-dependent representations must be learnt from limited training data.	Parallel-jaw finger	[29]
GANs used a single RGB image to predict the hand’s shape and position for gripping multiple objects.	Unstable training required a careful tuning of the hyperparameters. Not well developed for body pose estimation. Grasp point was not selected on the basis of the desired action and the state of the object, which can affect the next iteration.	Multi-finger (Human hand)	[37]
An SRL was proposed based on the disentanglement of a raw input image.	Evaluated only on a virtual platform; difficulties arise in translating models learnt from simulated images into real-world photos, referred to as “reality gap”.	Parallel-jaw finger	[52]
An approach that used a learnt grasp sampler was proposed to predict the full 6D grasp pose and account for any undetectable parts due to occlusions in the clutter by learning to differentiate between successful grasps and grasps that collide with the environment. Segmented point clouds were used in this scheme.	Did not assess if the hand mesh was colliding, e.g., whether a grasp was in a collision; instead, another trained network was used to predict the potential collisions.	Parallel-jaw finger	[41]
The GenerAL approach was proposed for 6-DoF grasping by leveraging deep RL to directly output the final position and configuration of the fingers.	Certain failure situations in clutter situations due to the highly dense cluster where there is no space for the robot to put its fingers. Did not concentrate on establishing object-specific grasping. Required a simulation setup, and in most situations, an extensive parameter search to operate sufficiently. Limited to known object models and was computationally expensive, requiring tens of seconds to minutes to accomplish.	Test on a range of parallel-jaw and multi-finger robot hands.	[47]
GG-CNN was proposed to extract pixel-by-pixel the grasp quality from a depth image. Meanwhile, the optimal grasp was predicted by considering the position, angle and grasping width.	Failed to grasp objects due to inaccurate visual information, as the depth camera cannot adequately identify the objects in a high-clutter environment. Failed to execute grasping on a black or transparent object in the middle of a clutter. Inaccurate grasp width estimation, which occurred frequently on big and small objects, resulting in gripper collision (e.g., the issue is related to the predicted grasp on a curved surface that may cause the object to slide out of the gripper in some cases; collisions with nearby objects are the most prevalent failure mode when objects are densely packed together in the clutter).	Parallel-jaw finger	[49]
An end-to-end network (Contact-GraspNet) was proposed to produce the distribution of 6-DoF parallel-jaw grasps efficiently and automatically from a scene’s depth data in cluttered scenes whilst avoiding collisions by projecting the 6-DoF into a 4-Dof grasp representation, which was composed of 3-DoF grasp rotation and grasp width.	Unable to grasp thick objects because of the fixed grasp width. Grasp predictions were less reliable because of the discontinuous selection boundary. Low confidence in estimating the contact poses of small objects because of their small effect on the total loss.	Parallel-jaw finger	[53]
The CARP was proposed to learn to estimate the probabilities of a collision-free grasp position, thus substantially enhancing the grasping of objects in challenging situations. Deep NNs were fully trained in simulation by self-supervision.	Robot handshapes were not considered in the learning, thus limiting the robot’s adaptability to diverse robot hands. Tested using a parallel-jaw gripper, which is less complicated in terms of kinematics restriction than the multi-finger gripper; however, in a crowded environment, parallel-jaw grippers are incapable of completing a variety of grasps.	Parallel-jaw finger	[45]
Generative DDGC was proposed to generate a set of collision-free multi-finger grasps in cluttered scenes.	High-quality grasps produced by DDGC do not always give a successful grasp in reality.	Multi-finger	[48]

**Table 3:** Important related studies involving the synergy of two actions based on object manipulation in cluttered environments.

Ref.	Challenge	Method	Weakness	Gripper	Mechanism	Success Rate
[65]	Grasping of objects placed in well-organized shapes	Deep Q-learning	Failure in certain cases because the robot proceeded to push the entire pile of objects, causing the items to be pushed out of the robot’s workspace. Performing a push movement when it was not necessary, resulting in a series of grasping and pushing actions.	Parallel-jaw finger	Push-to-grasp	80.3%
[67]	Grasping of objects aligned with the bin wall or boundaries	Deep Q-learning	Low robustness associated with the lack of depth information from the stereo cameras (due to shadows or reflecting surfaces). Less effective when the robot dealt with deformable and fragile objects. Only handled a low-level degree of clutter because the robot focused on grasping of objects aligned with the bin wall or boundaries. Success rate was poor because the test situations varied considerably (e.g., from 77% to 100%).	Parallel-jaw finger	Shift-to-grasp	91.7%
[62][63][61]	Grasping of objects placed among highly random cluttered objects	DQN	The DQN produced ineffective pushing actions that did not affect the operation scene; this weakness was due to insufficient training cases, although such issues can be handled automatically by adding more training cases. Failure was caused by inappropriate selection of grasp point (e.g., at the object’s edge). Necessitates extensive training iterations, which is time consuming, in addition to more robust training to overcome the challenge of active affordance prediction.	Multifunctional gripper	Push-to-grasp	77%
[71]	Grasping of objects in well-organized shapes	Deep Q-learning	Pushing tactic with sparse rewards lacked relevance for enhancing the grasping objective. The test scenarios in the randomly cluttered challenge did not indicate the level of clutter, which contradicted their method’s performance in the arranged object challenge.	Parallel-jaw finger	Push-to-grasp	83.1%
[72]	Grasping of objects in cluttered bins	Deep Q-learning	Suction grasp required a more precise and robust grasp point, which necessitated more data. Suction grasp failed when the object was partially covered by other items, implying that the grasp pose was lacking, and thus caused a failure in grasping. In the context of existing data, data were sparsely labeled as merely a single pixel; data collection was limited.	Suction cup	Poke-to-grasp	N/A
[66]	Grasping of objects in well-organized shapes	The twin delayed deep deterministic policy gradient	Push movements were performed unnecessarily, resulting in a series of grasping and pushing actions.	Parallel-jaw finger	Push-to-grasp	73.5%
[69]	Grasping of objects placed randomly in clutters	Attention DQN	Push movements were performed unnecessarily, resulting in a series of grasping and pushing actions. The DQN produced ineffective pushing actions that did not affect the operation scene. Their test scenarios in the randomly cluttered challenge did not indicate the level of clutter (e.g., maximum number of objects is only 20), and the push performance was not evaluated with the arranged object challenge.	Parallel-jaw finger	Push-to-grasp	73.5%
[68]	Grasping of objects aligned with the bin wall or boundaries	Deep Q-learning	Their test scenarios in the randomly cluttered challenge did not indicate the level of clutter, and the push performance was not evaluated with the arranged object challenge. Push movements were performed unnecessarily, resulting in a series of grasping and pushing actions.	Parallel-jaw finger	Push-to-grasp	74.6%
[78]	Grasping of objects in well-organised shapes	A duelling-DDQN	Push movements were performed unnecessarily, resulting in a series of grasping and pushing actions due to the standard strategy denoted by the (*s*) = *max*[*Q_p_*,*Q_g_*]. Even if the environment changes throughout the execution process, the max *Q*-value could occasionally fail to determine the correct action to be executed.	Parallel-jaw finger	Push-to-grasp	94%

**Table 4:** Important articles on finding an object in a cluttered environment.

Ref	Method	Weakness	Gripper	Mechanism	Success Rate
[89]	Mask-RCNN was used to detect the target object, and then Q-learning was trained on two FCNs by using DenseNet (a pre-trained model) in parallel, in which the first learning estimated the push action direction, whereas the second learning determined the possible grasp point; this approach executed grasping and pushing based on the max-value generated by the depth heightmap’s heatmap	Assumed prior knowledge and relied on a predefined target object. The testing scenarios were concentrated on a single object type, which cannot be applied to other objects; unfamiliar objects required further training before they can be acknowledged. Max-value-based action selection, making a push movement when it was not required, resulting in a sequence of grasping and pushing actions.	Parallel-jaw finger	Push-to-grasp	N/A
[90]	Volumetric representation of a scene state by using TSDF and detections RL’s agent in PPO was used to coordinate the learning of grasping or pushing	Discretized representation to encode observation history, thus affecting the next iteration assumption given the need to ‘forget’ the volume where the objects had moved. Assumed prior knowledge and relied on a target object with a specific color (e.g., red cube); besides the red color, the model must be retrained. In the actual world, some end-effector positions are kinematically impossible to achieve. Their test scenarios had a lower level of clutter.	Parallel-jaw finger	Push-to-grasp	97.3%
[91]	A target-oriented method was proposed based on DQN and divided into two sections: exploration (finding the target object in the clutter, whether visible or not) and coordination (choosing the right action, such as pushing or grasping).	Assumed prior knowledge and relied on a target object with a specific color. The entire pushing reward function was built manually; may need numerous tuning iterations and lacks adaptability to new scenarios.	Parallel-jaw finger	Push-to-grasp	86.0%
[100]	Deep Q-learning	Assumed prior knowledge and relied on a target object with a specific color. Deep Q-learning is model-free and does not make predictions about future states.	Parallel-jaw finger	Push-to-grasp	83.1%
[101]	Quasi-static push and overhand grasp movements were used to extract a target object from a densely packed environment. The presented VFT method identified the shortest sequence of actions by using DIPN to estimate the push action outcomes. MCTS was used to predict and choose the best action.	The time required by VFT was long due to the large MCTS tree that must be computed. Assumed prior knowledge and relied on a target object with a specific color.	Parallel-jaw finger	Push-to-grasp	98.5%
[103]	The DQN-based Obstacle Rearrangement (DORE) algorithm	Their approach was limited to retrieving the target object using identical grids space-based performing rearrangement objects, which cannot be applied to instances that were not modeled by regularly spaced identical grids. It was unable to cope with the challenge of multiple objects in an environment where invisible space exists because of objects being occluded. Once the number of obstacles multiplied, their success rate dropped dramatically.	Parallel-jaw finger	Pick-to-place	74~95%

## Data Availability

The authors confirm that the data supporting the findings of this study are available within the article.
